# The Growing Importance of Mixed-Methods Research in Health

**DOI:** 10.3126/nje.v12i1.43633

**Published:** 2022-03-31

**Authors:** Sharada Prasad Wasti, Padam Simkhada, Edwin R. van Teijlingen, Brijesh Sathian, Indrajit Banerjee

**Affiliations:** 1,2 School of Human and Health Sciences, University of Huddersfield, United Kingdom; 3 Centre for Midwifery, Maternal and Perinatal Health, Bournemouth University, Bournemouth, United Kingdom; 4 Geriatrics and long term care Department, Rumailah Hospital, Hamad Medical Corporation, Doha, Qatar; 5 Sir Seewoosagur Ramgoolam Medical College, Belle Rive, Mauritius

**Keywords:** Research Design, Epidemiologic Study Characteristics, Qualitative Research

## Abstract

This paper illustrates the growing importance of mixed-methods research to many health disciplines ranging from nursing to epidemiology. Mixed-methods approaches requires not only the skills of the individual quantitative and qualitative methods but also a skill set to bring two methods/datasets/findings together in the most appropriate way. Health researchers need to pay careful attention to the ‘best’ approach to designing, implementing, analysing, integrating both quantitative (number) and qualitative (word) information and writing this up in a way offers greater insights and enhances its applicability. This paper highlights the strengths and weaknesses of mixed-methods approaches as well as some of the common mistakes made by researchers applying mixed-methods for the first time.

## Background

Quantitative and qualitative research methods each address different types of questions, collect different kinds of data and deliver different kinds of answers. Each set of methods has its own inherent strengths and weaknesses, and each offers a particular approach to address specific types of research questions (and agendas). Health disciplines such as dentistry, nursing, speech and language therapy, and physiotherapy often use either quantitative or qualitative research methods on their own. However, there is a steadily growing literature showing the advantages of mixed-methods research is used in the health care and health service field [1-2]. Although we have advocated the use of mixed-methods in this journal eight years ago [3], there is still not enough mixed-methods research training in the health research field, particularly for health care practitioners, such as nurses, physiotherapists, midwives, and doctors, wanting to do research. Mixed-methods research has been popular in the social sciences since the twentieth century [4], and it has been growing in popularity among healthcare professionals [5], although it is still underdeveloped in disciplines such nursing and midwifery [6,7].

### Underpinning philosophies

To help understand that mixed-methods research is not simply employing two different methods in the same study, one needs to consider their underpinning research philosophies (also called paradigms). First, quantitative research is usually underpinned by positivism. This includes most epidemiological studies; such research is typically based on the assumption that there is one single real world out there that can be measured. For example, quantitative research would address the question “What proportion of the population of India drinks coffee?” Secondly, qualitative research is more likely to be based on interpretivism. This includes research based on interviews and focus groups, research which us is typically based on the assumption that we all experience the world differently. Since we all live in a slightly different world in our heads the task of qualitative research is to analyse the interpretations of the people in the sample. For example, qualitative research would address the question “How do people experience drinking coffee in India?”, and “What does drinking coffee mean to them?”

Mixed-methods research brings together questions from two different philosophies in what is being referred to as the third path [8], third research paradigm [9,10], the third methodology movement [11,12] and pragmatism [5]. The two paradigms differ in key underlying assumptions that ultimately lead to choices in research methodology and methods and often give a breadth by answering more complicated research questions [4]. The roles of mixed-methods are clear in an understanding of the situation (the what), meaning, norms, values (the why or how) within a single research question which combine the strength of two different method and offer multiple ways of looking at the research question [13]. Epidemiology sits strongly in the quantitative research corner, with a strong emphasis on large data sets and sophisticated statistical analysis. Although the use of mixed methods in health research has been discussed widely researchers raised concerns about the explanation of why and how mixed methods are used in a single research question [5].

### The relevance of mixed-methods in health research

The overall goal of the mixed-methods research design is to provide a better and deeper understanding, by providing a fuller picture that can enhance description and understanding of the phenomena [4]. Mixed-methods research has become popular because it uses quantitative and qualitative data in one single study which provides stronger inference than using either approach on its own [4]. In other words, a mixed-methods paper helps to understand the holistic picture from meanings obtained from interviews or observation to the prevalence of traits in a population obtained from surveys, which add depth and breadth to the study. For example, a survey questionnaire will include a limited number of structured questions, adding qualitative methods can capture other unanticipated facets of the topic that may be relevant to the research problem and help in the interpretation of the quantitative data. A good example of a mixed-methods study, it one conducted in Australia to understand the nursing care in public hospitals and also explore what factors influence adherence to nursing care [14]. Another example is a mixed-methods study that explores the relationship between nursing care practices and patient satisfaction. This study started with a quantitative survey to understand the general nursing services followed by qualitative interviews. A logistic regression analysis was performed to quantify the associations between general nursing practice variables supplemented with a thematic analysis of the interviews [15]. These research questions could not be answered if the researchers had used either qualitative or quantitative alone. Overall, this fits well with the development of evidence-based practice.

Despite the strengths of mixed-methods research but there is not much of it in nursing and other fields [7]. A recent review paper shows that the prevalence of mixed-methods studies in nursing was only 1.9% [7]. Similarly, a systematic review synthesised a total of 20 papers [16], and 16 papers [17] on nursing-related research paper among these only one mixed-methods paper was identified. Worse, a further two mixed-methods review recently revealed that out of 48 [18,19] synthesised nursing research papers, not one single mixed-methods paper was identified. This clearly depicts that mixed-methods research is still in its infancy stage in nursing but we can say there is huge scope to implement it to understand research questions on both sides of coin [4]. Therefore, there is a great need for mixed-methods training to enhance the evidence-based decision making in health and nursing practices.

### Strengths and weaknesses of mixed-methods

There are several challenges in identifying expertise of both methods and in working with a multidisciplinary, interdisciplinary, or transdisciplinary team [20]. It increases costs and resources, takes longer to complete as mixed-methods design often involves multiple stages of data collection and separate data analysis [4,5]. Moreover, conducting mixed-methods research does not necessarily guarantee an improvement in the quality of health research. Therefore, mixed-methods research is only appropriate when there are appropriate research questions [4,6].

Identifying an appropriate mixed-methods journal can also be challenging when writing mixed-methods papers [21]. Mixed-methods papers need considerably more words than single-methods papers as well as sympathetic editors who understand the underlying philosophy of a mixed-methods approach. Such papers, simply require more words. The mixed-methods researcher must be reporting two separate methods with their own characteristics, different samples, and ways of analysing, therefore needs more words to describe both methods as well as both sets of findings. Researcher needs to find a journal that accepts longer articles to help broaden existing evidence-based practice and promote its applicability in the nursing field [22].

### Common mistakes in applying mixed-methods

Not all applied researchers have insight into the underlying philosophy and/or the skills to apply each set of methods appropriately. Younas and colleagues’ review identified that around one-third (29%) of mixed-methods studies did not provide an explicit label of the study design and 95% of studies did not identify the research paradigm [7]. Whilst several mixed-methods publications did not provide clear research questions covering both quantitative and qualitative approaches. Another common issue is how to collect data either concurrent or sequential and the priority is given to each approach within the study where equal or dominant which are not clearly stated in writing which is important to mention while writing in the methods section. Similarly, a commonly overlooked aspect is how to integrate both findings in a paper. The responsibility lies with the researcher to ensure that findings are sufficiently plausible and credible [4]. Therefore, intensive mixed-methods research training is required for nursing and other health practitioners to ensure its appropriate.

### The way forward

Despite the recognised strengths and benefits of doing mixed-methods research, there is still only a limited number of nursing and related-health research publications using such this approach. Researchers need training in how to design, conduct, analyse, synthesise and disseminate mixed-methods research. Most importantly, they need to consider appropriate research questions that can be addressed using a mixed methods approach to add to our knowledge in evidence-based practice. In short, we need more training on mixed-methods research for a range of health researchers and health professionals.
